# A Multi-Center, Randomized, Double-Blind Placebo-Controlled Trial of Intravenous-Ibuprofen (IV-Ibuprofen) for Treatment of Pain in Post-Operative Orthopedic Adult Patients

**DOI:** 10.1111/j.1526-4637.2010.00896.x

**Published:** 2010-08

**Authors:** Neil Singla, Amy Rock, Leo Pavliv

**Affiliations:** * Lotus Clinical Research Inc.Pasadena, California; † Cumberland Pharmaceuticals Inc.Nashville, Tennessee, USA

**Keywords:** Ibuprofen, Pain, Analgesic, NSAID, Opioid, Post-Operative

## Abstract

**Objective:**

To determine whether pre- and post-operative administration of intravenous ibuprofen (IV-ibuprofen) can significantly decrease pain and morphine use when compared with placebo in adult orthopedic surgical patients.

**Design:**

This was a multi-center, randomized, double-blind placebo-controlled trial.

**Setting:**

This study was completed at eight hospitals; six in the United States and two in South Africa.

**Patients:**

A total of 185 adult patients undergoing elective orthopedic surgery.

**Interventions:**

Patients were randomized to receive either 800 mg IV-ibuprofen or placebo every 6 hours, with the first dose administered pre-operatively. Additionally, all patients had access to intravenous morphine for rescue.

**Outcome Measures:**

Efficacy of IV-ibuprofen was demonstrated by measuring the patient's self assessment of pain using a visual analog scale (VAS; assessed with movement and at rest) and a verbal response scale (VRS). Morphine consumption during the post-operative period was also assessed.

**Results:**

In the immediate post-operative period, there was a 25.8% reduction in mean area under the curve-VAS assessed with movement (AUC-VASM) in patients receiving IV-ibuprofen (*P* < 0.001); a 31.8% reduction in mean AUC-VAS assessed at rest (AUC-VASR; *P* < 0.001) and a 20.2% reduction in mean VRS (*P* < 0.001) compared to those receiving placebo. Patients receiving IV-ibuprofen used 30.9% less morphine (*P* < 0.001) compared to those receiving placebo. Similar treatment emergent adverse events occurred in both study groups and there were no significant differences in the incidence of serious adverse events.

**Conclusion:**

Pre- and post-operative administration of IV-ibuprofen significantly reduced both pain and morphine use in orthopedic surgery patients in this prospective randomized placebo-controlled trial.

## Introduction

Opioids are considered the treatment cornerstone for severe post-operative pain [1]. In the United States, more than 60% of the patients who have experienced moderate or severe post-operative pain have received morphine as a post-operative pain therapy [2]. A multimodal approach to pain management is an effective means of achieving comprehensive pain relief, while minimizing the potential for opioid-related adverse effects by lowering the total dose of opioid that is required [3].

A previous randomized, placebo controlled trial in surgical patients, including those undergoing orthopedic procedures, evaluated pain reduction and the opioid sparing effect of intravenous ibuprofen (IV-ibuprofen) during multimodal post-operative pain management [4]. This trial demonstrated that 800 mg IV-ibuprofen dosed every 6 hours produced a significant reduction in pain and morphine use in surgical patients [4].

## Methods

This was a multi-center, randomized, double-blind, placebo-controlled study of the safety and efficacy of IV-ibuprofen for post-operative pain following elective knee or hip replacement, reconstruction or arthroplasty with an anticipated need for post-operative hospitalization and IV morphine analgesia for at least 28 hours. This study was approved by institutional review boards or independent ethics committees at all participating sites. Efficacy was measured by comparing the patients' self-assessment of pain and morphine use in the post-surgical period.

### Study Design

Patients who met all of the inclusion criteria and did not meet any exclusion criteria (Table 1) were consented and randomized in a 1:1 ratio (treatment assignment was blinded) to receive either 800 mg of IV-ibuprofen or placebo (visually indistinguishable). The 800 mg dose, and the dosing schedule (every 6 hours), was selected based on results from previous clinical studies of IV-ibuprofen for the management of post-operative pain in hospitalized patients [4].

**Table 1:** Inclusion and exclusion criteria

Inclusion Criteria
1.Scheduled for elective hip or knee replacement, reconstruction or arthroplasty surgery with anticipated need for post-operative I.V. morphine analgesia with anticipated use of >28 hours.
2.Adequate IV access
3.Anticipated hospital stay >28 hours
Exclusion Criteria
1.Be unable to make a reliable self-report of pain intensity to pain relief
2.Less than 18 years of age
3.Greater than 80 years of age
4.Use of analgesics, muscle relaxants, NSAIDs and sedatives less than 12 hours prior to CTM administration with the following exceptions: acetaminophen (paracetamol) can be administered until 6 hours prior to surgery, tramadol can be administered until midnight the evening prior to surgery, muscle relaxants working at the neuromuscular junction used for intubation and/or anesthesia administration for the surgical procedure prior to CTM administration, and sedatives (i.e., midazolam) used as a co-induction agent for the surgical procedure prior to CTM administration
5.Patients taking warfarin, lithium, combination of ACE-inhibitors and furosemide
6.Patients with anemia (active, clinically significant anemia) and/or a history or evidence of asthma or heart failure
7.History of allergy or hypersensitivity to any component of intravenous ibuprofen, aspirin (or aspirin related products), NSAIDs, or COX-2 inhibitors
8.Pregnant or nursing
9.History of severe head trauma that required current hospitalization, intracranial surgery or stroke within the previous 30 days, or any history of intracerebral arteriovenous malformation, cerebral aneurism or CNS mass lesion
10.Weigh less than 30 kg
11.Have a history of congenital bleeding diathesis (e.g., hemophilia) or any active clinically significant bleeding, or have underlying platelet dysfunction including (but not limited to) idiopathic thrombocytopenic purpura, disseminated intravascular coagulation, or congenital platelet dysfunction
12.Have GI bleeding that required medical intervention within the previous 6 weeks (unless definitive surgery has been performed)
13.Have a platelet count less than 30,000 mm^3^ determined within the 28 days prior to surgery
14.Pre-existing dependence on narcotics or known tolerance to opioids
15.Inability to understand the requirements of the study, be willing to provide written informed consent (as evidenced by signature on an informed consent document approved by an Institutional Review Board [IRB]), and agree to abide by the study restrictions and to return for the required assessments
16.Refusal to provide written authorization for use and disclosure of protected health information
17.Be on dialysis, have oliguria or creatinine > 3.0 mg/dL
18.Inability to achieve hemostasis or inability to close surgical incision, prior to operating room discharge
19.Operative procedure includes organ transplant
20.Pre or intra-operative procedure utilized for the prevention of pre- or post-operative pain (i.e., epidural or nerve blocks)
21.Be receiving full dose anticoagulation therapy or Activated Protein C within 6 hours before dosing (Prophylaxis with subcutaneous heparin is acceptable)
22.Have received another investigational drug within the past 30 days
23.Be otherwise unsuitable for the study in the opinion of the investigator

* Use of anticoagulants for prevention of DVT's was permitted.

NSAID = nonsteroidal anti-inflammatory drug; CTM = clinical trial material; ACE = angiotensin-converting enzyme; COX-2 = cyclooxygenase 2; CNS = central nervous system.

In previous clinical studies of IV-ibuprofen, the initial dose was administered intra-operatively. However, the current trial sought to determine if IV-ibuprofen would also have a significant effect on the onset of pain. Therefore, the first dose of study drug was administered prior to the surgical procedure. The study drug was infused over 30 minutes through either a peripheral or a central venous catheter. After surgery, all patients had access to PCA morphine.

Previous clinical studies demonstrated that despite having access to morphine, patients receiving IV-ibuprofen not only used less morphine, but also reported lower pain scores [4]. The placebo control was chosen to accurately assess the treatment effect associated with IV-ibuprofen, utilizing both pain scores and the morphine-sparing model.

### Patient Population and Interventions

The trial was conducted in an in-patient setting at a total of eight surgical hospital sites. Six of the eight sites were in the United States: Dekalb Medical Center (Decatur, GA), Arkansas Surgical Hospital (North Little Rock, AR), Foundation Surgical Hospital (Bellaire, TX), Memorial Hermann-Memorial City Hospital (Houston, TX), Methodist Hospital (Arcadia, CA), and Springhill Memorial Hospital (Mobile, AL). Two of the eight sites were in South Africa: Johannesburg General Hospital (Johannesburg, Gauteng, RSA) and Eugene Marias Hospital (Pretoria, Gauteng, RSA). Enrollment was open to adults between ages 18 and 80 years who were scheduled for elective hip or knee replacement, reconstruction or arthroplasty, with an anticipated hospital stay and anticipated concomitant need for IV morphine analgesia for at least 28 hours post-procedure. Patients were required to weigh more than 30 kg, have adequate venous access as well as the ability to perform a pain intensity report, and not have a narcotic dependence or a known tolerance to opioids (Table 1).

**Figure 1:**
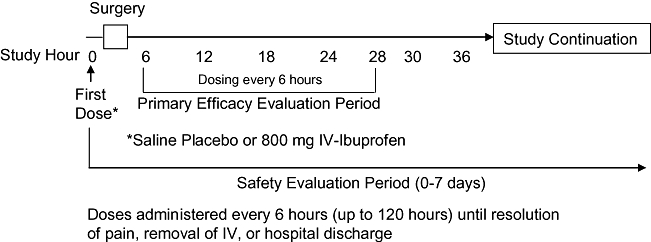
Study timeline.

Patients were randomized to receive either 800 mg of IV ibuprofen or placebo and the clinical trial duration was 7 days (Figure 1). The first dose of study drug was administered at the induction of anesthesia, prior to the surgical procedure (Figure 1). Four subsequent doses of study drug were to be administered every 6 hours over the next 24 hours (Figure 1). After the patient received five scheduled doses of study drug, additional doses could be administered as needed every 6 hours through the 120-hour treatment period (Figure 1).

Upon discharge from the operating room, all patients had access to intravenous morphine via a PCA pump. Initial settings were a 1 mg demand dose with a 5-minute lockout. If adequate pain control was not achieved the demand dose of morphine could be increased to 2 mg every 5 minutes, and additional morphine could be administered at the treating physician's discretion. However, basal infusions of morphine were not allowed. A data safety monitoring committee reviewed all serious adverse events reported during the study.

All patients received general anesthesia using various anesthetics such as propofol or a member of the flurane drug family along with fentanyl for intraoperative narcotic maintenance (fentanyl dose was per the discretion of the anesthesiologist). Intraoperative use of any other analgesics including nonsteroidal agents was prohibited. Neuraxial anesthesia (local anesthetics and narcotics) were prohibited. Single shot and catheter-based regional techniques were also prohibited.

### Efficacy Assessments

The primary objective of this study was to determine the efficacy of IV-ibuprofen compared with placebo for the treatment of surgical pain during hours 6–28 post-operatively, demonstrated by patient self-assessment of pain with movement (VASM), using a visual analog scale (VAS). Secondary objectives included several efficacy measurements to help elucidate the analgesic effect of perioperative IV ibuprofen in the 6–28 hour time period including 1) the patients' self-assessment of pain at rest measured by the VAS assessed at rest (VASR), 2) pain measured by the verbal rating scale (VRS), and 3) differences in post-operative morphine consumption between groups.

Prior to the scheduled 6-hour post-operative pain assessment, a VASM and VASR were performed as soon as the patient was awake and alert enough to properly perform the procedure. On average, patients were able to perform this assessment at 2.81 hours post-operatively. An additional secondary endpoint (deemed early post-op pain) examined the differences between treatment groups at this time.

Pain assessments were performed at the following time points: immediately following surgery as soon as the patient was awake enough to comply with study procedures, at hours 6, 8, 12, 16, 20, 24, 28, and then 2 hours after any subsequent doses of study medication that was administered in the 28–72 hours post-operative period. Additionally, after the 28-hour mark, scheduled VAS scores were obtained each day until the study drug was discontinued.

The VAS used was a standard 100-mm scale. VRS pain assessment measured a patient's self-report of pain on a numerical scale from 0 (no pain) to 4 (severe pain at rest and with movement). Additional measurements of efficacy included in the study were incidence of treatment failure, time to GI motility, time to resumption of ambulation, time to resumption of liquid intake and solid diet, incidence of opioid related side effects, and length of hospital stay.

### Safety Assessments

The safety profile of 800 mg IV-ibuprofen infused every 6 hours vs placebo was evaluated through comparison of treatment-emergent adverse events, as well as vital signs, clinical chemistry values, hematology and coagulation values, and transfusion requirements. Investigators were asked to report any adverse medical events (new events or worsening conditions) that occurred during the seven day study and to grade the event by intensity (mild, moderate, severe), record action taken with the study drug, causality to the study drug, required treatment, outcome, and time of resolution. Vital signs and laboratory results were monitored closely to investigate the effects of treatment, including renal-related effects and any possible impact on bleeding, during screening, at baseline, at hours 1, 3, 6, 9, 12, 24 hours after study drug administration, and daily through day 5 or discharge. Requirements for transfusions of blood products (including packed red blood cells, fresh frozen plasma, and platelets) were also collected. Clinical laboratory assessments included clinical chemistry (sodium, potassium, chloride, total carbon dioxide, glucose, blood urea nitrogen, creatinine, total bilirubin, albumin, total protein, aspartate aminotransferase, alanine aminotransferase, lactate dehydrogenase). Hematology and coagulation assessments included white blood cell count and differential, hematocrit, hemoglobin, platelet count, activated partial thromboplastin time, and prothrombin time or INR. Lab results were assessed during screening, baseline, and on study days 1, 2, 3, and 5 or at discharge. Adverse event monitoring continued through day 14. In the event that a patient was discharged before the end of the study, safety assessments were conducted by follow-up visit or telephone.

Patients were also evaluated with a combined safety assessment: 1) occurrence of diffuse pruritus; 2) occurrence of overt respiratory depression; 3) need for post-operative urinary indwelling catheter (after initial removal of surgical catheter); 4) incidence of post-operative vomiting or need for anti-emetic medication; and 5) the Richmond Agitation Sedation Scale. The initial Combined Safety Assessment was performed following surgery upon reversal of anesthesia, and then at post-operative hours 6, 8, 12, 16, 20, 24, and 28.

### Analysis Methods

The study population consisted of patients who were randomized and received at least one dose of study medication.

A literature review of morphine dosing shows that there is a correlation between dose and patient age and weight. In an attempt to ensure a more balanced overall randomization, treatment groups were stratified by age (≤45 and >45–80 years) and weight (≤75 kg and >75 kg). Patients greater than 80 years of age were excluded to reduce variability because these patients generally require significantly less morphine for pain management [5]. Data obtained from an earlier study on IV-ibuprofen were used in the sample size considerations. A sample size of 67 per group provided 90% power to detect a 20% reduction in pain-area under the curve (AUC) with movement with a significance level (alpha) of 0.05 using a two-sided two-sample (independent) *t*-test. Sample size estimates were done using NCSS/PASS software (NCSS, Kaysville, UT) [6]. Analysis of the primary efficacy parameters was conducted on the study population.

The AUC from 6 to 28 hours for the VAS assessed with movement (AUC-VASM [6–28]) was the primary efficacy endpoint, and the analysis of variance (anova) was applied. The primary model included factors for study center, treatment group, age group, weight group and their interactions. Interactions that were not statistically significant at the 20% level were removed from the model before computing the test for treatment difference, while interactions that were statistically significant at the 20% level were retained as alpha-level adjustments [7].

Frequency and percent of treatment failure was compared across treatment groups using a Chi-square test, while the time to treatment failure was evaluated using survival methods. Median time to treatment failure was calculated using the Kaplan–Meier approach and the treatment groups were compared with a log-rank test.

Computation of the AUC measures utilized all available information. Exact times of observations were imputed whenever available. Since the trapezoidal rule was used to compute the AUC, any absent scheduled observations were estimated by linear interpolation from the observations on either side of the missing data point, utilizing direct-likelihood mixed model repeated measures methodology [8].

Adverse events were coded using MedDRA preferred terminology and MedDRA system organ classifications, and the adverse events were also coded as serious or non-serious using FDA definitions.

## Results

A total of 185 patients were enrolled at eight clinical sites in the United States and South Africa. Table 2 presents the demographics and characteristics for the study population (all randomized patients who received at least one dose of study medication). The mean age for all patients was 61 (±10.2 SD) years, and the ages ranged from a minimum of 24 to a maximum of 80 years. There was no difference in age between treatment groups: 800 mg IV-ibuprofen 62 (±10.3 SD); placebo 60 (±9.9 SD).

**Table 2:** Demographics and baseline characteristics

	Placebo	Intravenous ibuprofen	Total
	n = 86	n = 99	n = 185
Age			
Mean (SD)	60 (9.9)	62 (10.3)	61 (10.2)
Age category			
<45 years old	6 (7%)	3 (3%)	9 (5%)
45–80 years old	80 (93%)	96 (97%)	176 (95%)
Gender			
Male	31 (36%)	34 (34%)	65 (35%)
Female	55 (64%)	65 (66%)	120 (65%)
Race			
Caucasian	60 (70%)	76 (77%)	136 (74%)
Black	24 (28%)	19 (19%)	43 (23%)
Hispanic	1 (1%)	3 (3%)	4 (2%)
Asian	0	1 (1%)	1 (<1%)
Other	1 (1%)	0	1 (<1%)
Height (cm)			
Mean (SD)	168.0 (11.18)	167.5 (10.22)	167.7 (10.65)
Weight (kg)			
Mean (SD)	96.6 (23.81)	89.2 (20.78)	92.6 (22.49)
Surgical procedure			
Hip replacement	14 (16%)	26 (26%)	40 (22%)
Hip reconstruction	0	0	0
Hip arthroplasty	5 (6%)	11 (11%)	16 (9%)
Knee replacement	50 (58%)	42 (42%)	92 (50%)
Knee reconstruction	0	0	0
Knee arthroplasty	17 (20%)	19 (19%)	36 (19%)
Other: surgery cancelled	0	1 (1%)	1 (<1%)

Of the 185 patients enrolled, 65 (35%) were male and 120 (65%) were female. There was no difference in gender between treatment groups. In the study population, 136 participants (74%) were Caucasian, 43 (23%) were Black, 4 (2%) were Hispanic, 1 (<1%) was Asian, and 1 (<1%) indicated “Other” for race.

The mean height for all patients was 167.7 cm (±10.7 SD), and there was no difference in height between treatment groups. The mean weight for all patients was 92.6 kg (±22.5 SD). Weight ranged from a minimum of 55.0 kg to a maximum of 183.0 kg. There was a significant difference in weight between treatment groups: 800 mg IV-ibuprofen 89.2 (±20.8 SD); placebo 96.6 (±23.8 SD) (*P* = 0.025).

Of the 185 patients enrolled, 92 (50%) underwent a knee replacement, 40 (22%) underwent a hip replacement, 36 (19%) underwent a knee reconstruction, and 16 (9%) underwent a hip reconstruction and one (<1%) had the surgery cancelled.

Clinical centers were asked to perform a baseline physical examination and to indicate whether body systems were normal or abnormal. Overall, the participants in this study had few abnormalities at baseline, except for the indication for surgery.

There was no difference in the number of doses administered between the IV-ibuprofen and the placebo treatment groups, with a median of five doses (range = 1 to 13 doses). The mean number of doses for the IV-ibuprofen treatment group was 6 doses (±SD 2.0), and 5 doses (±SD 1.4) for the placebo treatment group.

### Efficacy

**Figure 2:**
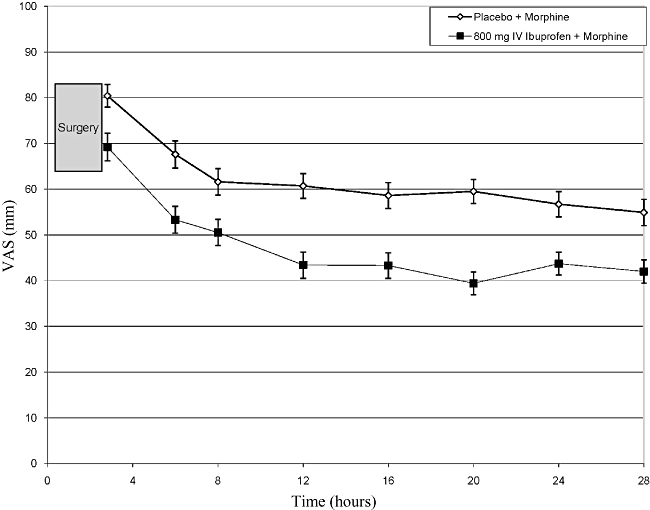
VASM scores, over time, post surgery-pain with movement was assessed by VAS at the first immediate post-operative opportunity (mean 2.81 hours) out to study hour 28 in the IV-ibuprofen and placebo treatment groups. Error bars denote SEM.

**Figure 3:**
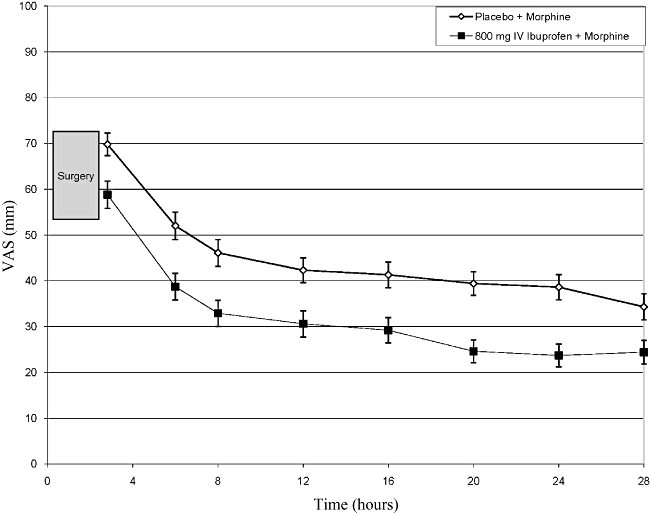
VASR scores, over time, post surgery-pain at rest was assessed by VAS at the first immediate post-operative opportunity (mean 2.81 hours) out to study hour 28 in the IV-ibuprofen and placebo treatment groups. Error bars denote SEM.

The primary objective of this study was to determine the efficacy of IV-ibuprofen compared with placebo for the treatment of post-operative surgical pain during hours 6–28 demonstrated by patient self-assessment of pain with movement, using a VAS. During the 6–28-hour post-operative period, when compared to patients receiving placebo, patients receiving IV-ibuprofen experienced:

- 25.8% decrease in the mean AUC-VASM (*P* < 0.001) (Figure 2 and Table 3).
- 31.8% decrease in the mean AUC-VASR (*P* < 0.001) (Figure 3 and Table 3).
- 20.2% decrease in the mean AUC-VRS (*P* < 0.001) (Table 4).
- 30.9% decrease in mean morphine consumption (*P* < 0.001) (Table 5).

**Table 3:** Summary of Pain Measured by VAS (AUC-VAS; with movement and at rest)

AUC	Placebo + Morphine (n = 86)	800 mg + Morphine (n = 99)
With movement		
6–28 hours (post-operative period)		
Mean (SD)	1,307.8 (388.7)	970.1 (422.2)
LS Means (SE)	1,326.1 (82.0)	1,005.0 (81.5)
Median	1,304.6	946.2
LS means difference (95% CI)		−321.1 (−436.7, −205.4)
*P* value vs Placebo*		<0.001
At rest		
6–28 hours (post-operative period)		
Mean (SD)	910.9 (424.3)	620.8 (401.0)
LS means (SE)	997.0 (83.6)	728.0 (83.0)
Median	905.1	566.0
LS means difference (95% CI)		−269.0 (−386.8, −151.2)
*P* value vs Placebo*		<0.001

* The analysis is based on a linear ANCOVA model with fixed effects for age group, weight group, randomization center, and treatment group. The *P* values and 95% confidence intervals are based on the difference in the LS Means from the final ANCOVA model. LS = least squares; VAS = visual analog scale; ANCOVA = analysis of covariance; AUC = area under the curve.

**Table 4:** Summary of pain measured by verbal response scale

AUC	Placebo (N = 80)	800 mg (N = 99)
6–28 hours (post-operative period)		
Mean (SD)	49.5 (18.2)	39.5 (17.1)
LS means (SE)	51.8 (3.7)	43.2 (3.6)
Median	50.7	38.0
LS means difference (95% CI)		−8.6 (−13.6, −3.6)
*P* value vs Placebo*		<0.001

* The analysis is based on a linear ANCOVA model with fixed effects for age group, weight group, randomization center, and treatment group. The p-values and 95% confidence intervals are based on the difference in the LS Means from the final ANCOVA model.

LS = least squares; AUC = area under the curve; ANCOVA = analysis of covariance.

**Table 5:** Summary of reduction in morphine consumption (mg morphine sulfate)

Morphine requirement	Placebo (N = 86)	800 mg (N = 99)
6–28 hours (post-operative period)		
N	85	97
Mean (SD)	59.5 (29.9)	41.1 (27.3)
LS means (SE)	60.9 (5.7)	44.3 (5.7)
Median	58.0	38.0
LS means difference (95% CI)		−16.6 (−24.7, −8.4)
*P* value vs Placebo*		<0.001

* The analysis is based on linear ANCOVA model with fixed effects for age group, weight group, randomization center, and treatment group. The p-values and 95% confidence intervals are based on the difference in the LS Means from the final ANCOVA model.

LS = least squares; ANCOVA = analysis of covariance.

The secondary endpoint evaluating early post-operative pain revealed that patients receiving a pre-operative dose of IV-ibuprofen experienced a 13.9% decrease in the mean VASM (*P* = 0.003) and a 15.8% decrease in the mean VASR (*P* = 0.012) following surgery (mean 2.81 hours after surgery) when compared with placebo (Table 6 and Figures 2 and 3).

**Table 6:** Summary of pain measured by VAS in immediate post-OP period

VAS-immediately post-surgery	Placebo (N = 86)	800 mg (N = 99)
With movement		
N	83	96
Mean (SD)	80.4 (22.4)	69.2 (29.3)
LS means (SE)	84.4 (5.2)	73.4 (5.0)
Median	88.0	73.5
LS means difference (95% CI)		−11.0 (−18.2, −3.8)
*P* value vs Placebo*		0.003
At rest		
N	83	96
Mean (SD)	69.8 (29.0)	58.8 (31.5)
LS means (SE)	75.3 (6.0)	64.6 (5.8)
Median	79.0	57.0
LS means difference (95% CI)		−10.7 (−19.0, −2.4)
*P* value vs Placebo*		0.012

* The analysis is based on a linear ANCOVA model with fixed effects for age group, weight group, randomization center, and treatment group. The p-values and 95% confidence intervals are based on the difference in the LS Means from the final ANCOVA model.

LS = least squares; ANCOVA = analysis of covariance.

Additional measurements of efficacy included the incidence of treatment failure, time to GI motility, time to resumption of ambulation, time to resumption of liquid intake and solid diet, incidence of opioid related side effects, and length of hospital stay. Due to the small sample size, there were no significant differences between treatment groups for any of these secondary efficacy measurements.

Efficacy data were collected past study hour 28; however, more than 80% of the patients had discontinued study medication by the 38 hour time point making any efficacy comparisons for the later time points statistically underpowered. Patients discontinued the study medication when their IV was removed or they were converted to oral analgesics.

### Safety

All 185 patients who were enrolled in the study and received at least one dose of study medication were included in the safety analysis population. As was previously mentioned, there was no difference in the number of doses administered between the IV-ibuprofen and the placebo groups. All patients had study medication stopped prior to treatment day 5, primarily due to the IV access being discontinued.

Treatment-emergent adverse events (AEs) and serious adverse events (SAES) were reported in 164 (89%) of the 185 patients (Table 7). In the IV-ibuprofen group, 90 of 99 (91%) patients experienced treatment-emergent AEs; 45 were of mild intensity, 39 were of moderate intensity, and 6 were severe. In the placebo group, 74 of 86 (86%) patients experienced treatment-emergent AEs; 36 were of mild intensity, 37 were of moderate intensity, and 1 was severe. There was not a statistically significant difference between treatment groups (*P* = 0.356).

**Table 7:** Treatment emergent adverse events and the types of adverse events that differed significantly between treatment groups

	Placebo (n = 86)		800 mg IV-Ibuprofen (n = 99)		Safety population (n = 185)		*P* value
Number of patients experiencing at least one adverse event	74 (86%)		90 (91%)		164 (89%)		0.356
Adverse events that differed significantly between treatment groups							
Type of adverse event	n	%	n	%	N	%	
Vomiting	12	(14%)	27	(27%)	39	(21%)	0.031
Dyspepsia	4	(5%)	0	0	4	(2%)	0.045

Nine subjects (5%) experienced a total of 11 SAEs. The relationship of SAEs to study drug was coded by the investigators as related, not related, and unknown or uncertain. In the IV-ibuprofen group, 6 of 99 (6%) participants experienced eight SAEs. In the placebo group, 3 of 86 (3%) participants experienced three serious adverse events. Of these 11 serious events, none were coded as related; four were coded as not related; and seven as unknown or uncertain. There was not a statistically significant difference in the number of patients experiencing SAEs when comparing the IV-ibuprofen treated patients with the placebo treated patients (*P* = 0.507).

The packed red blood cell transfusions between study day 0 and 5 did not differ between the IV-ibuprofen group, with 12 of 99 (12%) requiring transfusions, and the placebo group, with 9 of 86 (10%) patients receiving packed red blood cells. There were no patient deaths during the 14 day study. While patients were excluded if they were receiving full dose anticoagulation therapy, approximately 19% of the patients enrolled in the study were receiving partial anticoagulation therapy in the 12 hours preceding surgery and 60% received anticoagulation therapy following surgery.

AEs and clinical laboratory assessments commonly associated with oral ibuprofen were specifically examined, including hypertension, bleeding and bruising related events (even if not the primary surgical site), and abnormal laboratory measurements. In AEs experienced by at least three subjects, there were no clinically significant differences in the incidence of known AEs associated with oral ibuprofen in the IV-ibuprofen group when compared to placebo. In the treatment-emergent AEs experienced by at least three patients, there were significantly more patients in the IV-ibuprofen group who experienced vomiting (*P* = 0.031), and significantly more patients in the placebo group who experienced dyspepsia (*P* = 0.045) (Table 7).

## Discussion

Pain is the greatest concern of patients before surgery [9]. Administered orally, ibuprofen is effective in blocking pain and inflammation, in part by preventing the production of prostaglandins. An intravenous formulation of ibuprofen has the potential to arrest the inflammatory cascade triggered by surgical procedures and to reduce or prevent the development of post-operative pain. A previous randomized, placebo controlled trial in surgical patients, including those undergoing orthopedic procedures, evaluated pain reduction and the opioid sparing effect of IV-ibuprofen during multimodal post-operative pain management and demonstrated that 800 mg IV-ibuprofen dosed every 6 hours produced a significant reduction in pain and morphine use in surgical patients [4]. This study involves the first report of pre-operative administration of IV-ibuprofen.

NSAIDs carry warnings for cardiovascular and gastrointestinal risk and should be used with caution in patients with congestive heart failure, renal impairment, a risk for blood clots, and/or a history of ulcers or gastrointestinal bleeding. The only other available injectable NSAID, ketorolac, includes additional warnings for use in geriatric patients and is contraindicated for use in children [10]. Additionally, it carries a black box warning against pre-operative administration, and according to the package insert, can only be administered for 5 days or less [10]. Therefore, the introduction of an NSAID that can be used safely prior to surgery adds another therapeutic modality to treat post-operative pain.

IV-ibuprofen, 800 mg administered every 6 hours starting with the onset of anesthesia in orthopedic surgery patients, reduced both pain and morphine consumption post-operatively. When compared with patients receiving placebo, patients receiving 800 mg IV-ibuprofen experienced less pain beginning in the early post-operative period and continuing at least through hour 28. There was a significant reduction in pain with movement and at rest with IV-ibuprofen as measured by the AUC-VAS during the 6–28-hour post-operative period. There was also a significant reduction in mean morphine consumption in the 6–28-hour post-operative period in patients receiving IV-ibuprofen.

The safety analysis encompassed all 185 patients that participated in the study, 164 of which experienced treatment emergent-AEs (serious and nonserious). There was no difference in the general incidence of AEs between the IV-ibuprofen and placebo treatment groups. Events related to bleeding and coagulation times, and transfusion requirements, did not differ between the two groups. There were no deaths reported in this study. There were a total of 11 SAEs occurring in nine patients, with no statistically significant difference in the incidence of SAEs between the patients receiving placebo and the patients receiving IV-ibuprofen. The SAEs were consistent with those typically observed in patients undergoing surgery and treated with anesthesia and morphine.

## Conclusions

Pre- and post-operative administration of IV-ibuprofen significantly reduced both pain and morphine use in orthopedic surgery patients in this prospective randomized controlled trial. The 800 mg dose provided patients with analgesic benefit and narcotic reduction. Post-operative pain control protocols can now consider inclusion of IV-ibuprofen as an option to offer patients a significant analgesic benefit while reducing the risks associated with morphine administration.
